# Supplementary material for: Targeting the p53/xCT/GSH Axis with PRIMA-1Met Combined with Sulfasalazine Shows Therapeutic Potential in Chronic Lymphocytic Leukemia
Source: Int J Mol Sci. 2025 Jun 10;26(12):5559. doi: 10.3390/ijms26125559 (PMC12193190; doi:10.3390/ijms26125559)
Supplement: Supplementary file 1 [file ijms-26-05559-s001.zip › ijms-3644269-supplementary.pptx]

## Slide 1
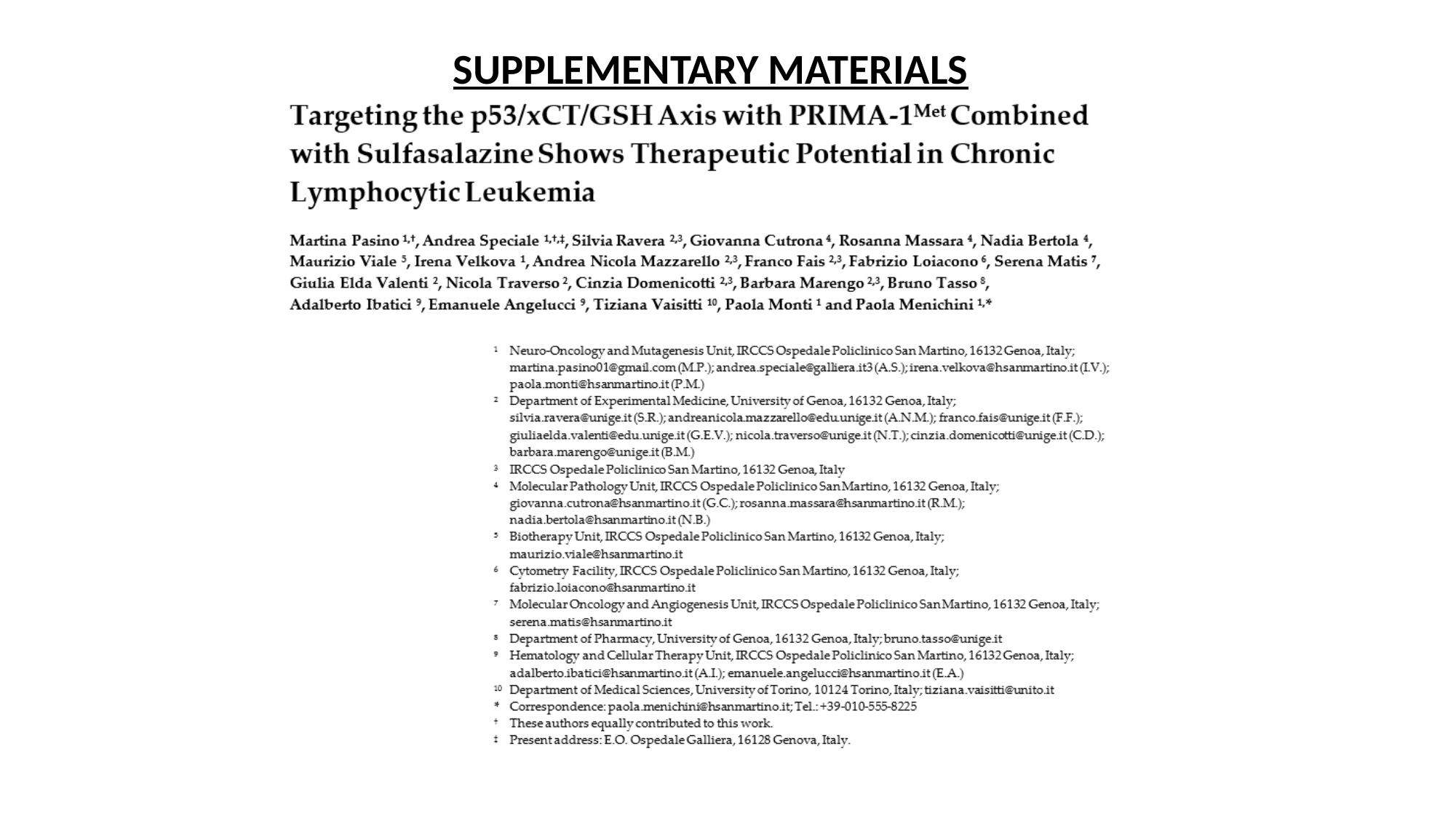

SUPPLEMENTARY MATERIALS

## Slide 2
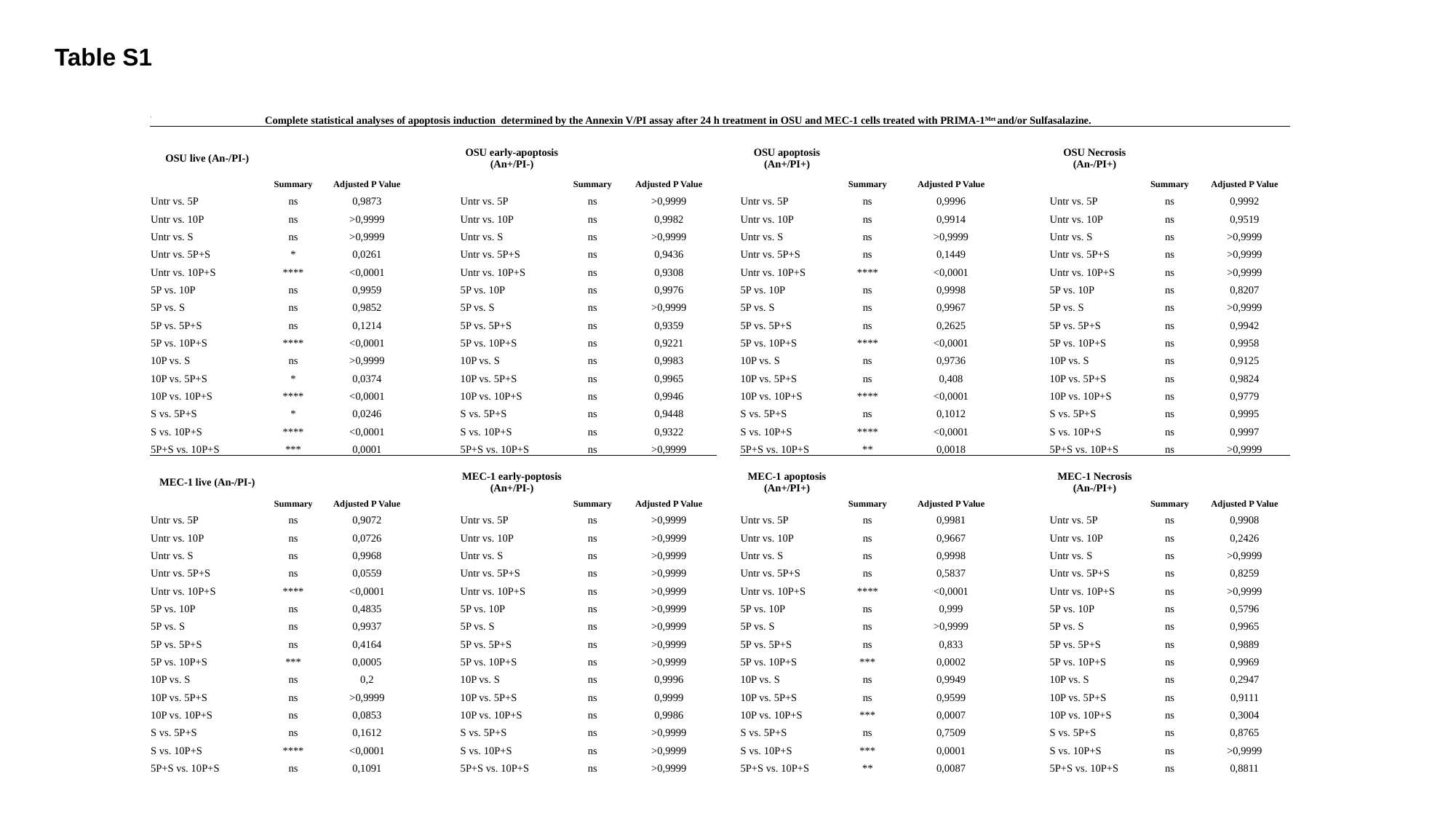

Table S1
| Table S1. | Complete statistical analyses of apoptosis induction determined by the Annexin V/PI assay after 24 h treatment in OSU and MEC-1 cells treated with PRIMA-1Met and/or Sulfasalazine. | | | | | | | | | | | | | |
| --- | --- | --- | --- | --- | --- | --- | --- | --- | --- | --- | --- | --- | --- | --- |
| OSU live (An-/PI-) | Summary | Adjusted P Value | | OSU early-apoptosis (An+/PI-) | Summary | Adjusted P Value | | OSU apoptosis (An+/PI+) | Summary | Adjusted P Value | | OSU Necrosis (An-/PI+) | Summary | Adjusted P Value |
| Untr vs. 5P | ns | 0,9873 | | Untr vs. 5P | ns | >0,9999 | | Untr vs. 5P | ns | 0,9996 | | Untr vs. 5P | ns | 0,9992 |
| Untr vs. 10P | ns | >0,9999 | | Untr vs. 10P | ns | 0,9982 | | Untr vs. 10P | ns | 0,9914 | | Untr vs. 10P | ns | 0,9519 |
| Untr vs. S | ns | >0,9999 | | Untr vs. S | ns | >0,9999 | | Untr vs. S | ns | >0,9999 | | Untr vs. S | ns | >0,9999 |
| Untr vs. 5P+S | \* | 0,0261 | | Untr vs. 5P+S | ns | 0,9436 | | Untr vs. 5P+S | ns | 0,1449 | | Untr vs. 5P+S | ns | >0,9999 |
| Untr vs. 10P+S | \*\*\*\* | <0,0001 | | Untr vs. 10P+S | ns | 0,9308 | | Untr vs. 10P+S | \*\*\*\* | <0,0001 | | Untr vs. 10P+S | ns | >0,9999 |
| 5P vs. 10P | ns | 0,9959 | | 5P vs. 10P | ns | 0,9976 | | 5P vs. 10P | ns | 0,9998 | | 5P vs. 10P | ns | 0,8207 |
| 5P vs. S | ns | 0,9852 | | 5P vs. S | ns | >0,9999 | | 5P vs. S | ns | 0,9967 | | 5P vs. S | ns | >0,9999 |
| 5P vs. 5P+S | ns | 0,1214 | | 5P vs. 5P+S | ns | 0,9359 | | 5P vs. 5P+S | ns | 0,2625 | | 5P vs. 5P+S | ns | 0,9942 |
| 5P vs. 10P+S | \*\*\*\* | <0,0001 | | 5P vs. 10P+S | ns | 0,9221 | | 5P vs. 10P+S | \*\*\*\* | <0,0001 | | 5P vs. 10P+S | ns | 0,9958 |
| 10P vs. S | ns | >0,9999 | | 10P vs. S | ns | 0,9983 | | 10P vs. S | ns | 0,9736 | | 10P vs. S | ns | 0,9125 |
| 10P vs. 5P+S | \* | 0,0374 | | 10P vs. 5P+S | ns | 0,9965 | | 10P vs. 5P+S | ns | 0,408 | | 10P vs. 5P+S | ns | 0,9824 |
| 10P vs. 10P+S | \*\*\*\* | <0,0001 | | 10P vs. 10P+S | ns | 0,9946 | | 10P vs. 10P+S | \*\*\*\* | <0,0001 | | 10P vs. 10P+S | ns | 0,9779 |
| S vs. 5P+S | \* | 0,0246 | | S vs. 5P+S | ns | 0,9448 | | S vs. 5P+S | ns | 0,1012 | | S vs. 5P+S | ns | 0,9995 |
| S vs. 10P+S | \*\*\*\* | <0,0001 | | S vs. 10P+S | ns | 0,9322 | | S vs. 10P+S | \*\*\*\* | <0,0001 | | S vs. 10P+S | ns | 0,9997 |
| 5P+S vs. 10P+S | \*\*\* | 0,0001 | | 5P+S vs. 10P+S | ns | >0,9999 | | 5P+S vs. 10P+S | \*\* | 0,0018 | | 5P+S vs. 10P+S | ns | >0,9999 |
| MEC-1 live (An-/PI-) | Summary | Adjusted P Value | | MEC-1 early-poptosis (An+/PI-) | Summary | Adjusted P Value | | MEC-1 apoptosis (An+/PI+) | Summary | Adjusted P Value | | MEC-1 Necrosis (An-/PI+) | Summary | Adjusted P Value |
| Untr vs. 5P | ns | 0,9072 | | Untr vs. 5P | ns | >0,9999 | | Untr vs. 5P | ns | 0,9981 | | Untr vs. 5P | ns | 0,9908 |
| Untr vs. 10P | ns | 0,0726 | | Untr vs. 10P | ns | >0,9999 | | Untr vs. 10P | ns | 0,9667 | | Untr vs. 10P | ns | 0,2426 |
| Untr vs. S | ns | 0,9968 | | Untr vs. S | ns | >0,9999 | | Untr vs. S | ns | 0,9998 | | Untr vs. S | ns | >0,9999 |
| Untr vs. 5P+S | ns | 0,0559 | | Untr vs. 5P+S | ns | >0,9999 | | Untr vs. 5P+S | ns | 0,5837 | | Untr vs. 5P+S | ns | 0,8259 |
| Untr vs. 10P+S | \*\*\*\* | <0,0001 | | Untr vs. 10P+S | ns | >0,9999 | | Untr vs. 10P+S | \*\*\*\* | <0,0001 | | Untr vs. 10P+S | ns | >0,9999 |
| 5P vs. 10P | ns | 0,4835 | | 5P vs. 10P | ns | >0,9999 | | 5P vs. 10P | ns | 0,999 | | 5P vs. 10P | ns | 0,5796 |
| 5P vs. S | ns | 0,9937 | | 5P vs. S | ns | >0,9999 | | 5P vs. S | ns | >0,9999 | | 5P vs. S | ns | 0,9965 |
| 5P vs. 5P+S | ns | 0,4164 | | 5P vs. 5P+S | ns | >0,9999 | | 5P vs. 5P+S | ns | 0,833 | | 5P vs. 5P+S | ns | 0,9889 |
| 5P vs. 10P+S | \*\*\* | 0,0005 | | 5P vs. 10P+S | ns | >0,9999 | | 5P vs. 10P+S | \*\*\* | 0,0002 | | 5P vs. 10P+S | ns | 0,9969 |
| 10P vs. S | ns | 0,2 | | 10P vs. S | ns | 0,9996 | | 10P vs. S | ns | 0,9949 | | 10P vs. S | ns | 0,2947 |
| 10P vs. 5P+S | ns | >0,9999 | | 10P vs. 5P+S | ns | 0,9999 | | 10P vs. 5P+S | ns | 0,9599 | | 10P vs. 5P+S | ns | 0,9111 |
| 10P vs. 10P+S | ns | 0,0853 | | 10P vs. 10P+S | ns | 0,9986 | | 10P vs. 10P+S | \*\*\* | 0,0007 | | 10P vs. 10P+S | ns | 0,3004 |
| S vs. 5P+S | ns | 0,1612 | | S vs. 5P+S | ns | >0,9999 | | S vs. 5P+S | ns | 0,7509 | | S vs. 5P+S | ns | 0,8765 |
| S vs. 10P+S | \*\*\*\* | <0,0001 | | S vs. 10P+S | ns | >0,9999 | | S vs. 10P+S | \*\*\* | 0,0001 | | S vs. 10P+S | ns | >0,9999 |
| 5P+S vs. 10P+S | ns | 0,1091 | | 5P+S vs. 10P+S | ns | >0,9999 | | 5P+S vs. 10P+S | \*\* | 0,0087 | | 5P+S vs. 10P+S | ns | 0,8811 |

## Slide 3
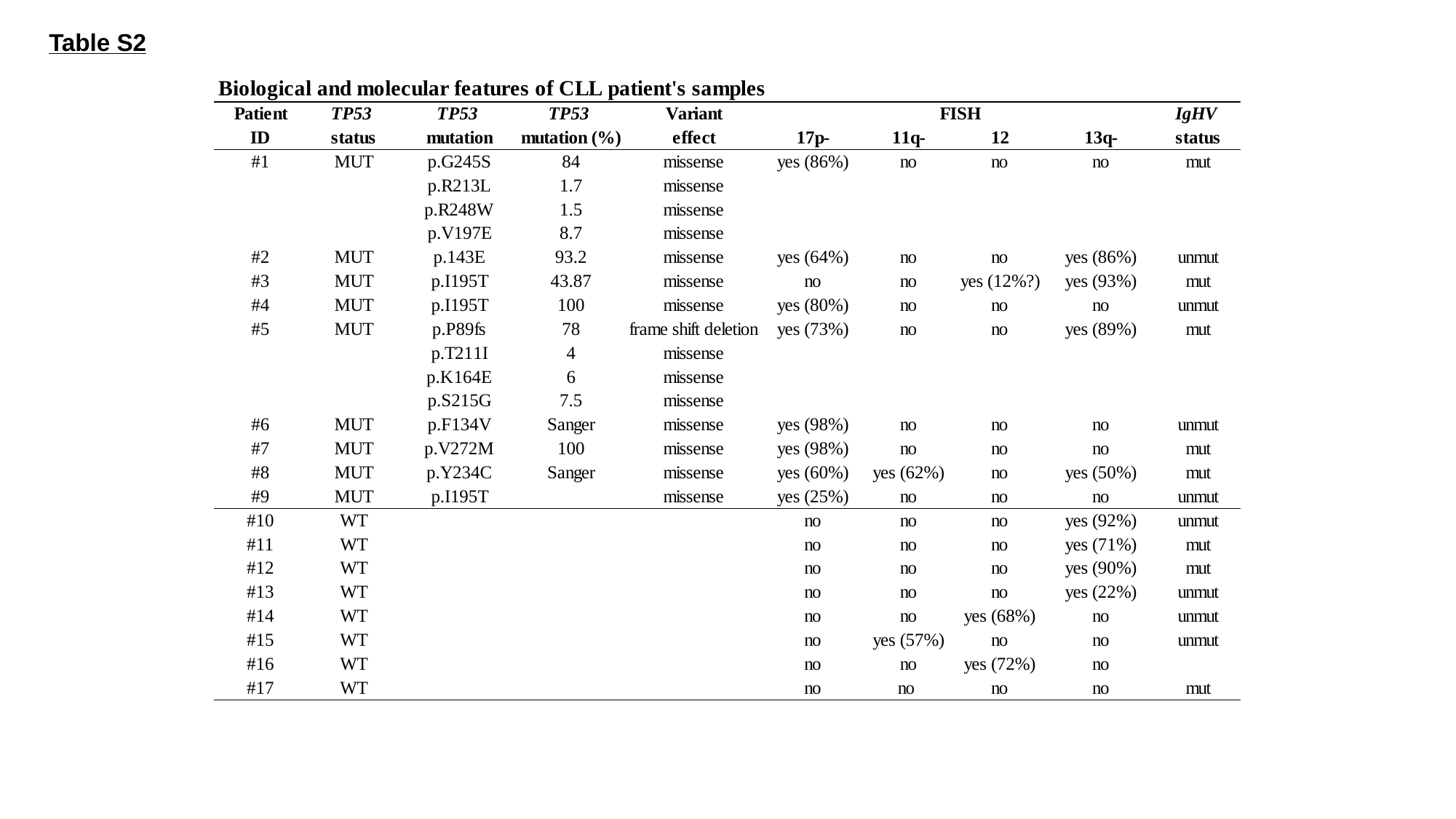

Table S2

## Slide 4
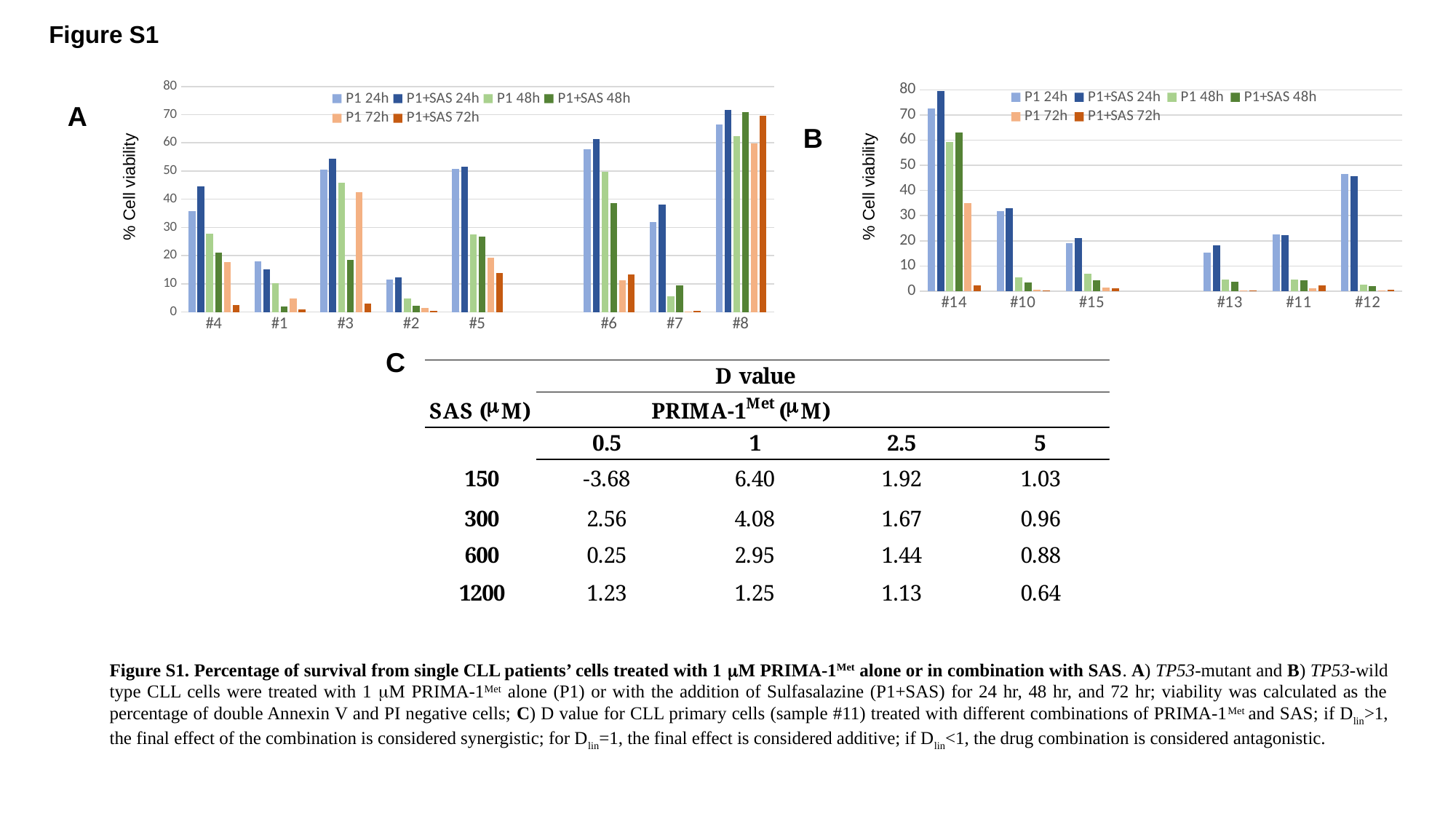

Figure S1
### Chart
| Category | P1 | P1+SAS 24h | P1 | P1+SAS 48h | P1 | P1+SAS 72h |
|---|---|---|---|---|---|---|
| #4 | 35.72 | 44.55 | 27.85 | 21.17 | 17.7 | 2.43 |
| #1 | 17.9 | 15.2 | 10.1 | 1.8 | 4.8 | 0.8 |
| #3 | 50.5 | 54.34 | 45.8 | 18.47 | 42.38 | 2.98 |
| #2 | 11.6 | 12.3 | 4.7 | 2.3 | 1.3 | 0.5 |
| #5 | 50.88 | 51.63 | 27.59 | 26.72 | 19.37 | 13.92 |
| | None | None | None | None | None | None |
| #6 | 57.61 | 61.41 | 49.66 | 38.55 | 11.16 | 13.18 |
| #7 | 31.9 | 38.0 | 5.5 | 9.5 | 0.1 | 0.4 |
| #8 | 66.4 | 71.8 | 62.5 | 70.8 | 59.7 | 69.7 |
### Chart
| Category | P1 | P1+SAS 24h | P1 | P1+SAS 48h | P1 | P1+SAS 72h |
|---|---|---|---|---|---|---|
| #14 | 72.6 | 79.4 | 59.4 | 63.0 | 34.9 | 2.2 |
| #10 | 31.85 | 33.02 | 5.53 | 3.33 | 0.44 | 0.27 |
| #15 | 18.99 | 20.96 | 7.05 | 4.38 | 1.32 | 1.07 |
| | None | None | None | None | None | None |
| #13 | 15.4 | 18.2 | 4.5 | 3.6 | 0.2 | 0.2 |
| #11 | 22.6 | 22.3 | 4.5 | 4.2 | 1.0 | 2.3 |
| #12 | 46.5 | 45.72 | 2.49 | 1.95 | 0.39 | 0.41 |A
B
% Cell viability
% Cell viability
C
Figure S1. Percentage of survival from single CLL patients’ cells treated with 1 mM PRIMA-1Met alone or in combination with SAS. A) TP53-mutant and B) TP53-wild type CLL cells were treated with 1 mM PRIMA-1Met alone (P1) or with the addition of Sulfasalazine (P1+SAS) for 24 hr, 48 hr, and 72 hr; viability was calculated as the percentage of double Annexin V and PI negative cells; C) D value for CLL primary cells (sample #11) treated with different combinations of PRIMA-1Met and SAS; if Dlin>1, the final effect of the combination is considered synergistic; for Dlin=1, the final effect is considered additive; if Dlin<1, the drug combination is considered antagonistic.

## Slide 5
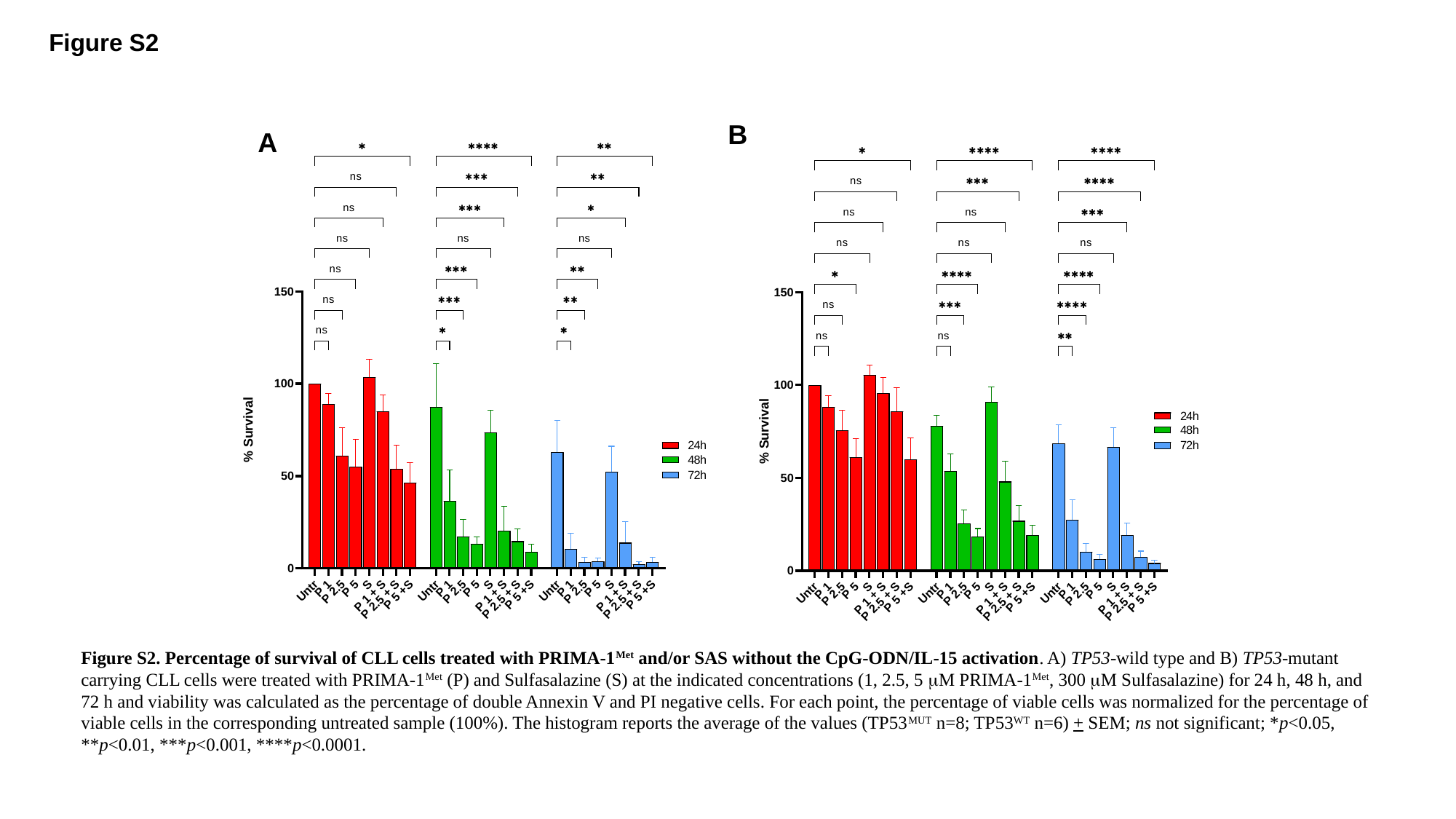

Figure S2
B
A
Figure S2. Percentage of survival of CLL cells treated with PRIMA-1Met and/or SAS without the CpG-ODN/IL-15 activation. A) TP53-wild type and B) TP53-mutant carrying CLL cells were treated with PRIMA-1Met (P) and Sulfasalazine (S) at the indicated concentrations (1, 2.5, 5 mM PRIMA-1Met, 300 mM Sulfasalazine) for 24 h, 48 h, and 72 h and viability was calculated as the percentage of double Annexin V and PI negative cells. For each point, the percentage of viable cells was normalized for the percentage of viable cells in the corresponding untreated sample (100%). The histogram reports the average of the values (TP53MUT n=8; TP53WT n=6) + SEM; ns not significant; *p<0.05, **p<0.01, ***p<0.001, ****p<0.0001.

## Slide 6
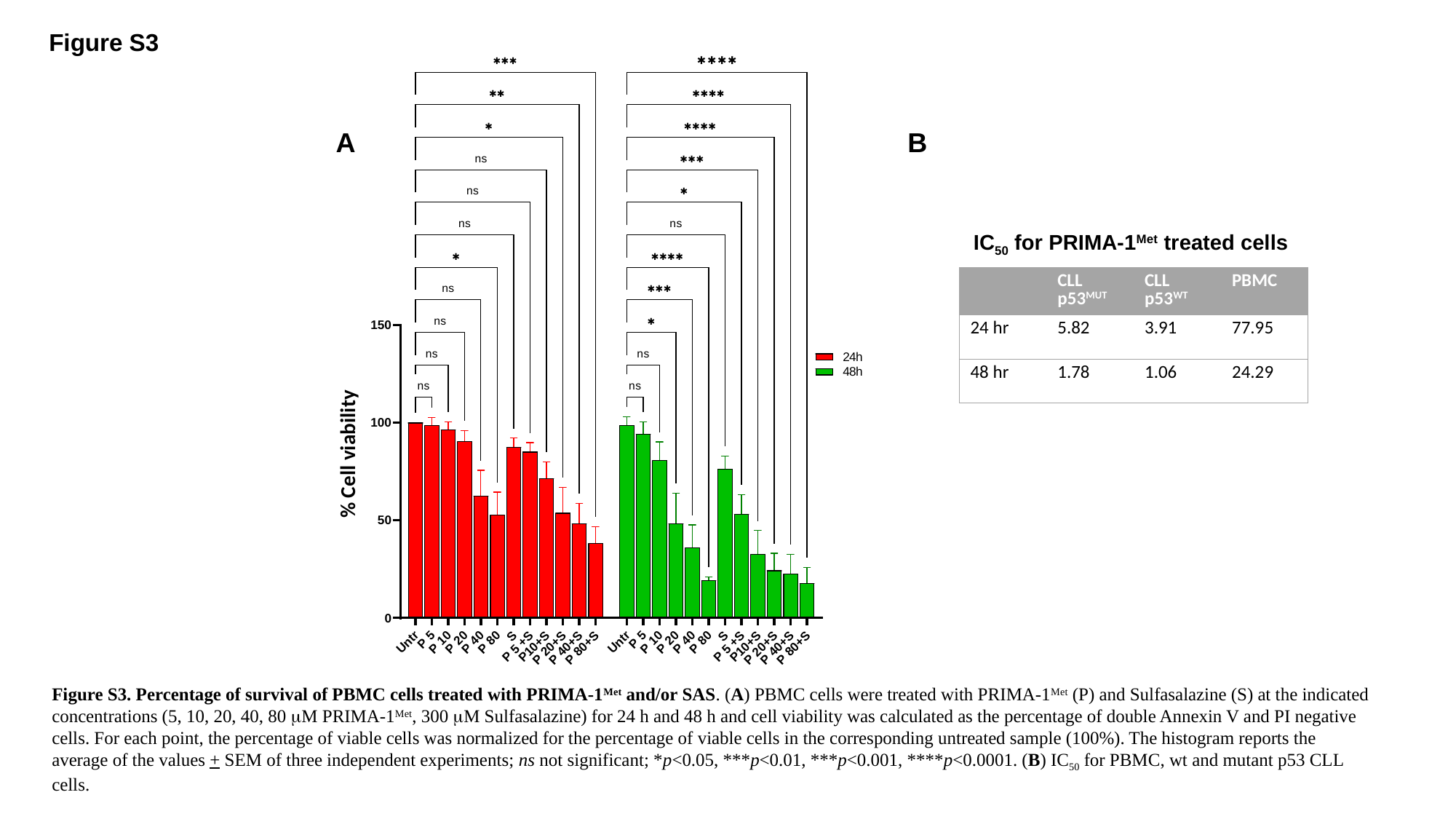

Figure S3
A
B
IC50 for PRIMA-1Met treated cells
| | CLL p53MUT | CLL p53WT | PBMC |
| --- | --- | --- | --- |
| 24 hr | 5.82 | 3.91 | 77.95 |
| 48 hr | 1.78 | 1.06 | 24.29 |
% Cell viability
Figure S3. Percentage of survival of PBMC cells treated with PRIMA-1Met and/or SAS. (A) PBMC cells were treated with PRIMA-1Met (P) and Sulfasalazine (S) at the indicated concentrations (5, 10, 20, 40, 80 mM PRIMA-1Met, 300 mM Sulfasalazine) for 24 h and 48 h and cell viability was calculated as the percentage of double Annexin V and PI negative cells. For each point, the percentage of viable cells was normalized for the percentage of viable cells in the corresponding untreated sample (100%). The histogram reports the average of the values + SEM of three independent experiments; ns not significant; *p<0.05, ***p<0.01, ***p<0.001, ****p<0.0001. (B) IC50 for PBMC, wt and mutant p53 CLL cells.

## Slide 7
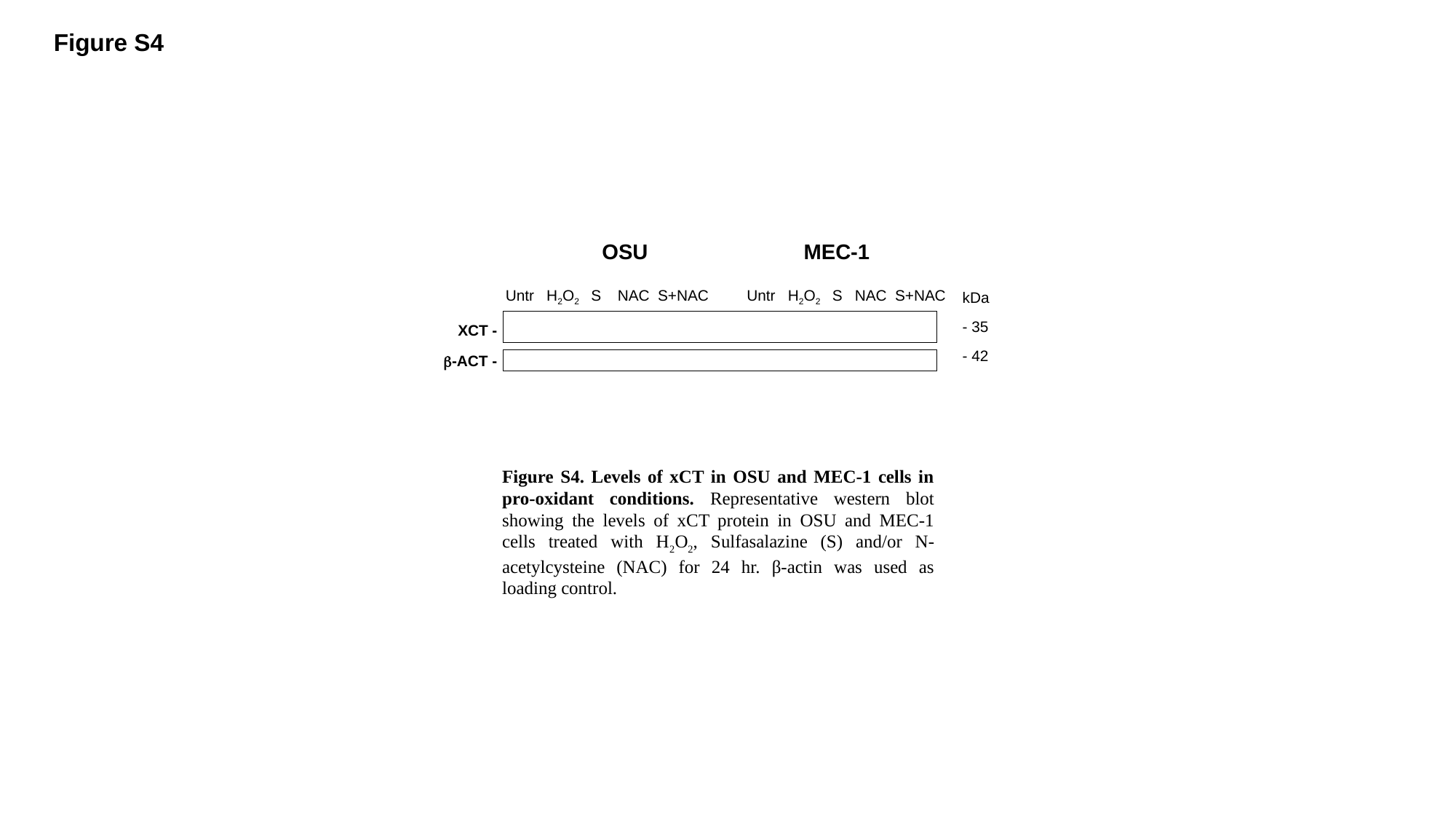

Figure S4
OSU
MEC-1
Untr H2O2 S NAC S+NAC
Untr H2O2 S NAC S+NAC
kDa
- 35
- 42
 XCT -
b-ACT -
Figure S4. Levels of xCT in OSU and MEC-1 cells in pro-oxidant conditions. Representative western blot showing the levels of xCT protein in OSU and MEC-1 cells treated with H2O2, Sulfasalazine (S) and/or N-acetylcysteine (NAC) for 24 hr. β-actin was used as loading control.

## Slide 8
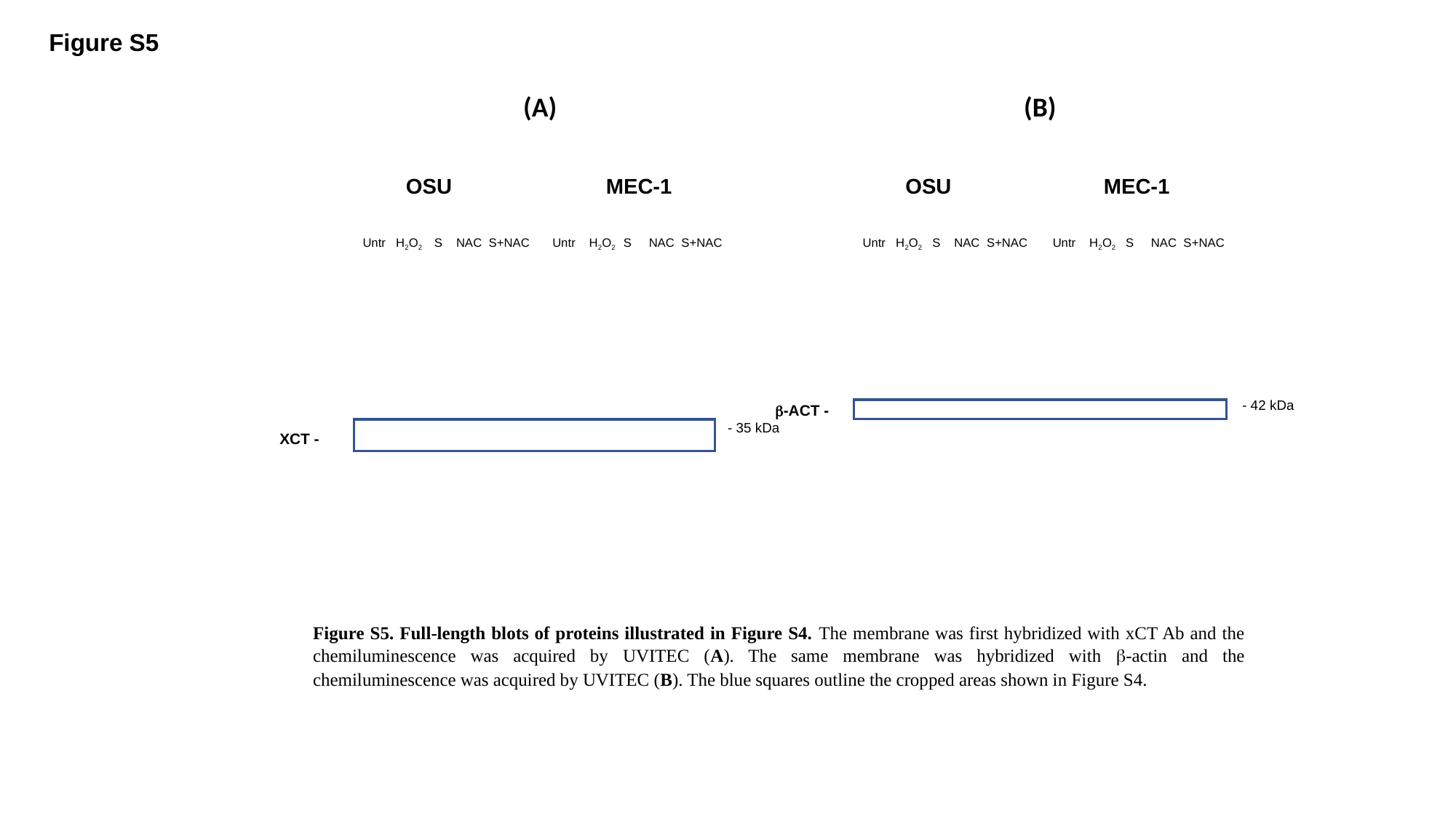

Figure S5
(A)
(B)
OSU
MEC-1
OSU
MEC-1
Untr H2O2 S NAC S+NAC
Untr H2O2 S NAC S+NAC
Untr H2O2 S NAC S+NAC
Untr H2O2 S NAC S+NAC
- 42 kDa
 b-ACT -
- 35 kDa
 XCT -
Figure S5. Full-length blots of proteins illustrated in Figure S4. The membrane was first hybridized with xCT Ab and the chemiluminescence was acquired by UVITEC (A). The same membrane was hybridized with b-actin and the chemiluminescence was acquired by UVITEC (B). The blue squares outline the cropped areas shown in Figure S4.

## Slide 9
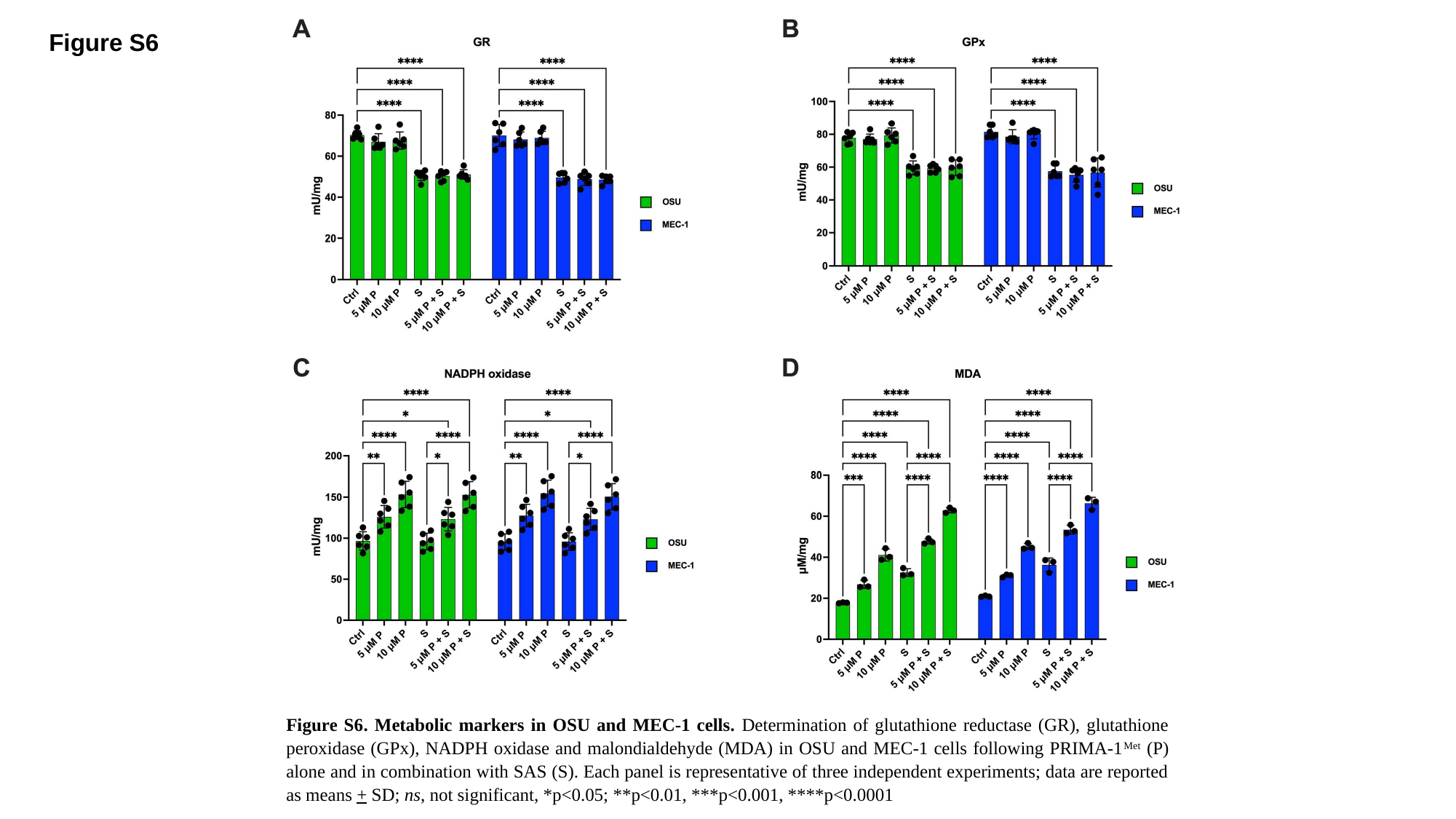

Figure S6
Figure S6. Metabolic markers in OSU and MEC-1 cells. Determination of glutathione reductase (GR), glutathione peroxidase (GPx), NADPH oxidase and malondialdehyde (MDA) in OSU and MEC-1 cells following PRIMA-1Met (P) alone and in combination with SAS (S). Each panel is representative of three independent experiments; data are reported as means + SD; ns, not significant, *p<0.05; **p<0.01, ***p<0.001, ****p<0.0001

## Slide 10
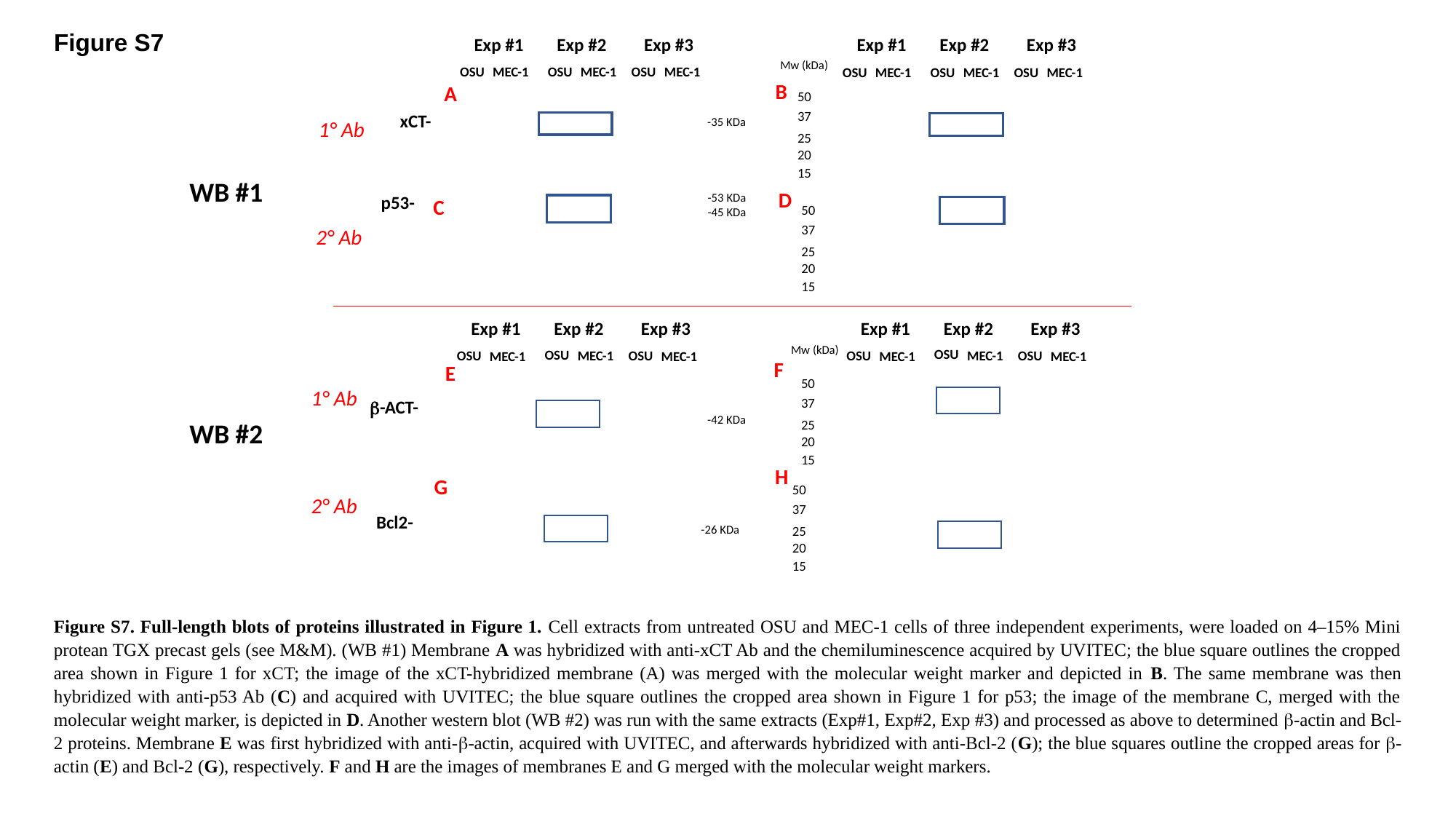

Figure S7
Exp #1 Exp #2 Exp #3
Exp #1 Exp #2 Exp #3
Mw (kDa)
OSU
MEC-1
OSU
MEC-1
OSU
MEC-1
OSU
MEC-1
OSU
MEC-1
OSU
MEC-1
B
A
50
37
25
20
15
xCT-
-35 KDa
1° Ab
WB #1
D
-53 KDa
-45 KDa
p53-
C
50
37
25
20
15
2° Ab
Exp #1 Exp #2 Exp #3
Exp #1 Exp #2 Exp #3
Mw (kDa)
OSU
OSU
OSU
MEC-1
OSU
OSU
MEC-1
OSU
MEC-1
MEC-1
MEC-1
MEC-1
F
E
50
37
25
20
15
1° Ab
b-ACT-
-42 KDa
WB #2
H
G
50
37
25
20
15
2° Ab
Bcl2-
-26 KDa
Figure S7. Full-length blots of proteins illustrated in Figure 1. Cell extracts from untreated OSU and MEC-1 cells of three independent experiments, were loaded on 4–15% Mini protean TGX precast gels (see M&M). (WB #1) Membrane A was hybridized with anti-xCT Ab and the chemiluminescence acquired by UVITEC; the blue square outlines the cropped area shown in Figure 1 for xCT; the image of the xCT-hybridized membrane (A) was merged with the molecular weight marker and depicted in B. The same membrane was then hybridized with anti-p53 Ab (C) and acquired with UVITEC; the blue square outlines the cropped area shown in Figure 1 for p53; the image of the membrane C, merged with the molecular weight marker, is depicted in D. Another western blot (WB #2) was run with the same extracts (Exp#1, Exp#2, Exp #3) and processed as above to determined b-actin and Bcl-2 proteins. Membrane E was first hybridized with anti-b-actin, acquired with UVITEC, and afterwards hybridized with anti-Bcl-2 (G); the blue squares outline the cropped areas for b-actin (E) and Bcl-2 (G), respectively. F and H are the images of membranes E and G merged with the molecular weight markers.

## Slide 11
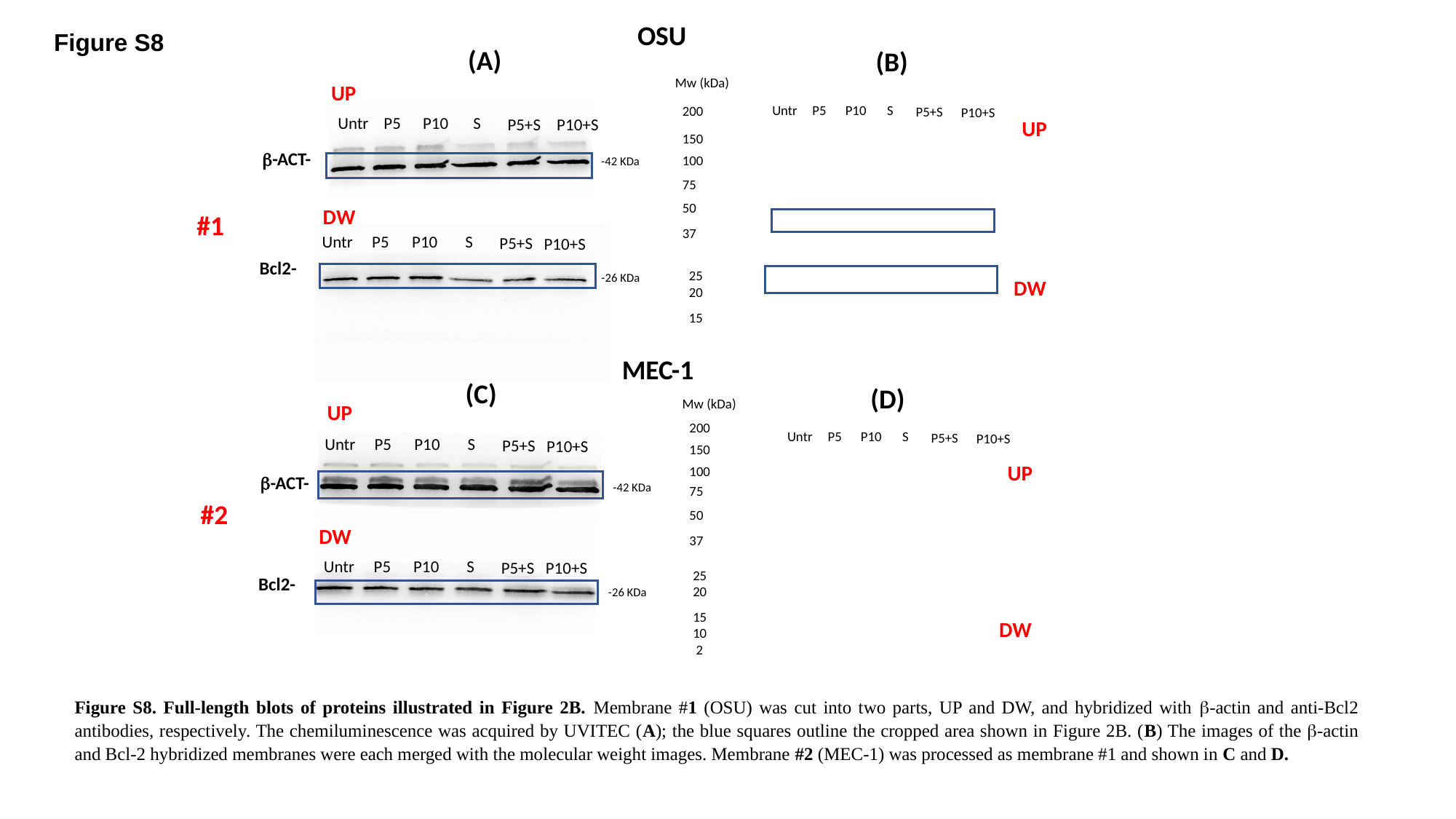

OSU
Figure S8
(A)
(B)
Mw (kDa)
UP
Untr
P5
P10
S
P5+S
P10+S
200
150
100
75
50
37
Untr
P5
P10
S
P5+S
P10+S
UP
b-ACT-
-42 KDa
DW
#1
Untr
P5
P10
S
P5+S
P10+S
Bcl2-
25
20
15
-26 KDa
DW
MEC-1
(C)
(D)
Mw (kDa)
UP
200
150
100
75
50
37
Untr
P5
P10
S
P5+S
P10+S
Untr
P5
P10
S
P5+S
P10+S
UP
b-ACT-
-42 KDa
#2
DW
Untr
P5
P10
S
P5+S
P10+S
25
20
15
10
 2
Bcl2-
-26 KDa
DW
Figure S8. Full-length blots of proteins illustrated in Figure 2B. Membrane #1 (OSU) was cut into two parts, UP and DW, and hybridized with b-actin and anti-Bcl2 antibodies, respectively. The chemiluminescence was acquired by UVITEC (A); the blue squares outline the cropped area shown in Figure 2B. (B) The images of the b-actin and Bcl-2 hybridized membranes were each merged with the molecular weight images. Membrane #2 (MEC-1) was processed as membrane #1 and shown in C and D.

## Slide 12
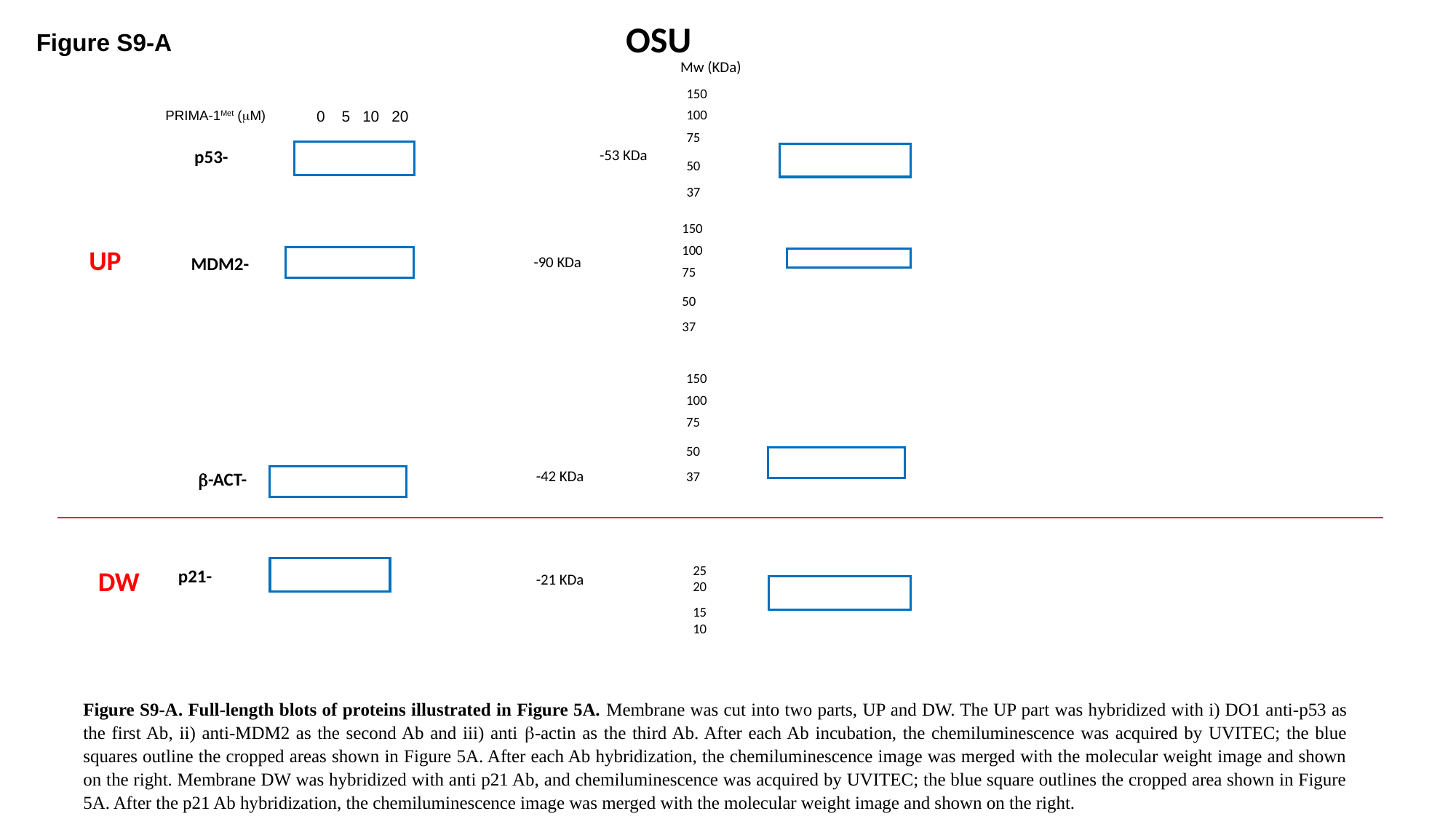

OSU
Figure S9-A
Mw (KDa)
150
100
75
50
37
PRIMA-1Met (mM)
0 5 10 20
p53-
-53 KDa
150
100
75
50
37
UP
MDM2-
-90 KDa
150
100
75
50
37
-42 KDa
b-ACT-
25
20
15
10
p21-
DW
-21 KDa
Figure S9-A. Full-length blots of proteins illustrated in Figure 5A. Membrane was cut into two parts, UP and DW. The UP part was hybridized with i) DO1 anti-p53 as the first Ab, ii) anti-MDM2 as the second Ab and iii) anti b-actin as the third Ab. After each Ab incubation, the chemiluminescence was acquired by UVITEC; the blue squares outline the cropped areas shown in Figure 5A. After each Ab hybridization, the chemiluminescence image was merged with the molecular weight image and shown on the right. Membrane DW was hybridized with anti p21 Ab, and chemiluminescence was acquired by UVITEC; the blue square outlines the cropped area shown in Figure 5A. After the p21 Ab hybridization, the chemiluminescence image was merged with the molecular weight image and shown on the right.

## Slide 13
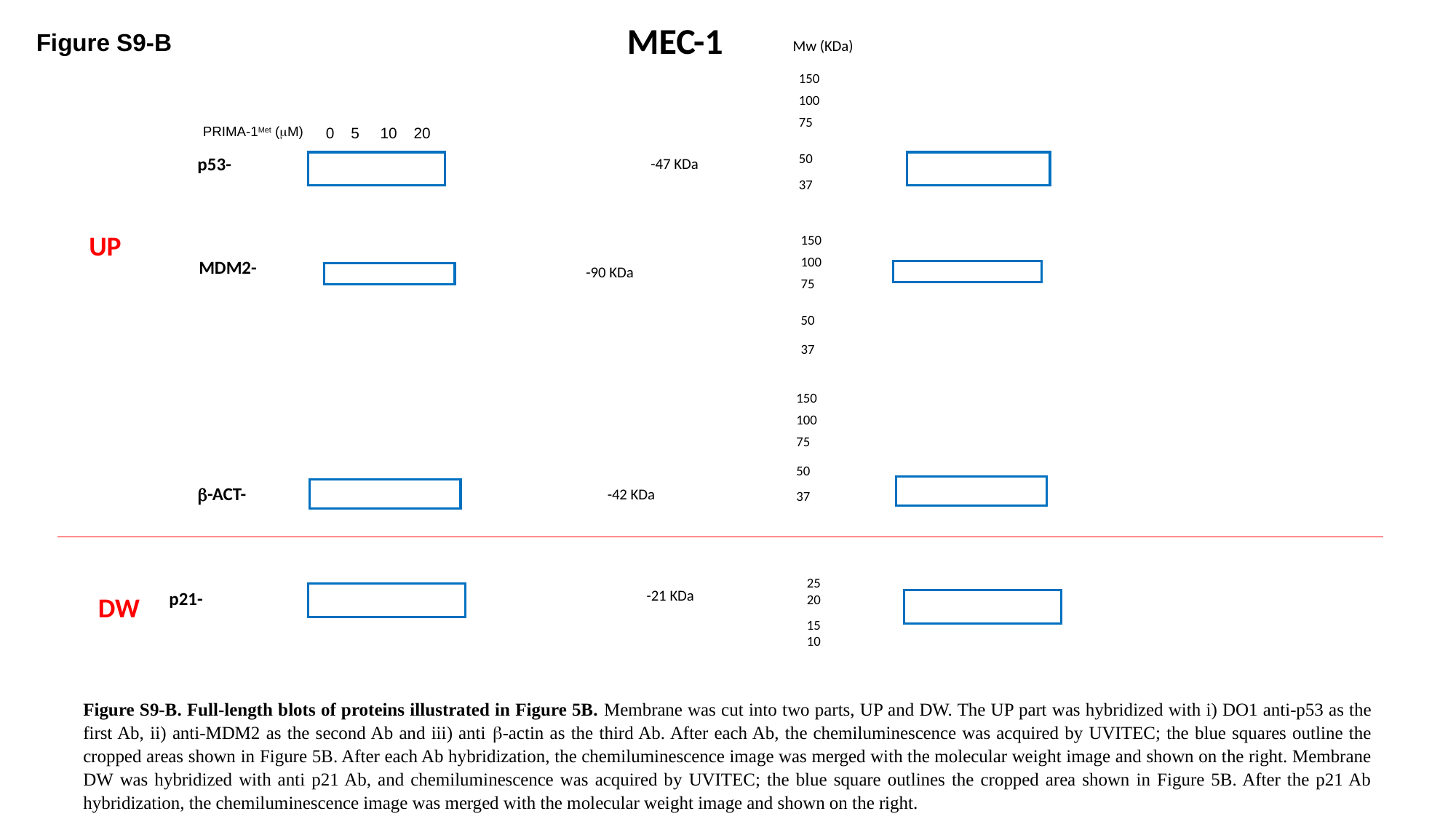

MEC-1
Figure S9-B
Mw (KDa)
150
100
75
50
37
PRIMA-1Met (mM)
0 5 10 20
p53-
-47 KDa
UP
150
100
75
50
37
MDM2-
-90 KDa
150
100
75
50
37
b-ACT-
-42 KDa
25
20
15
10
-21 KDa
p21-
DW
Figure S9-B. Full-length blots of proteins illustrated in Figure 5B. Membrane was cut into two parts, UP and DW. The UP part was hybridized with i) DO1 anti-p53 as the first Ab, ii) anti-MDM2 as the second Ab and iii) anti b-actin as the third Ab. After each Ab, the chemiluminescence was acquired by UVITEC; the blue squares outline the cropped areas shown in Figure 5B. After each Ab hybridization, the chemiluminescence image was merged with the molecular weight image and shown on the right. Membrane DW was hybridized with anti p21 Ab, and chemiluminescence was acquired by UVITEC; the blue square outlines the cropped area shown in Figure 5B. After the p21 Ab hybridization, the chemiluminescence image was merged with the molecular weight image and shown on the right.

## Slide 14
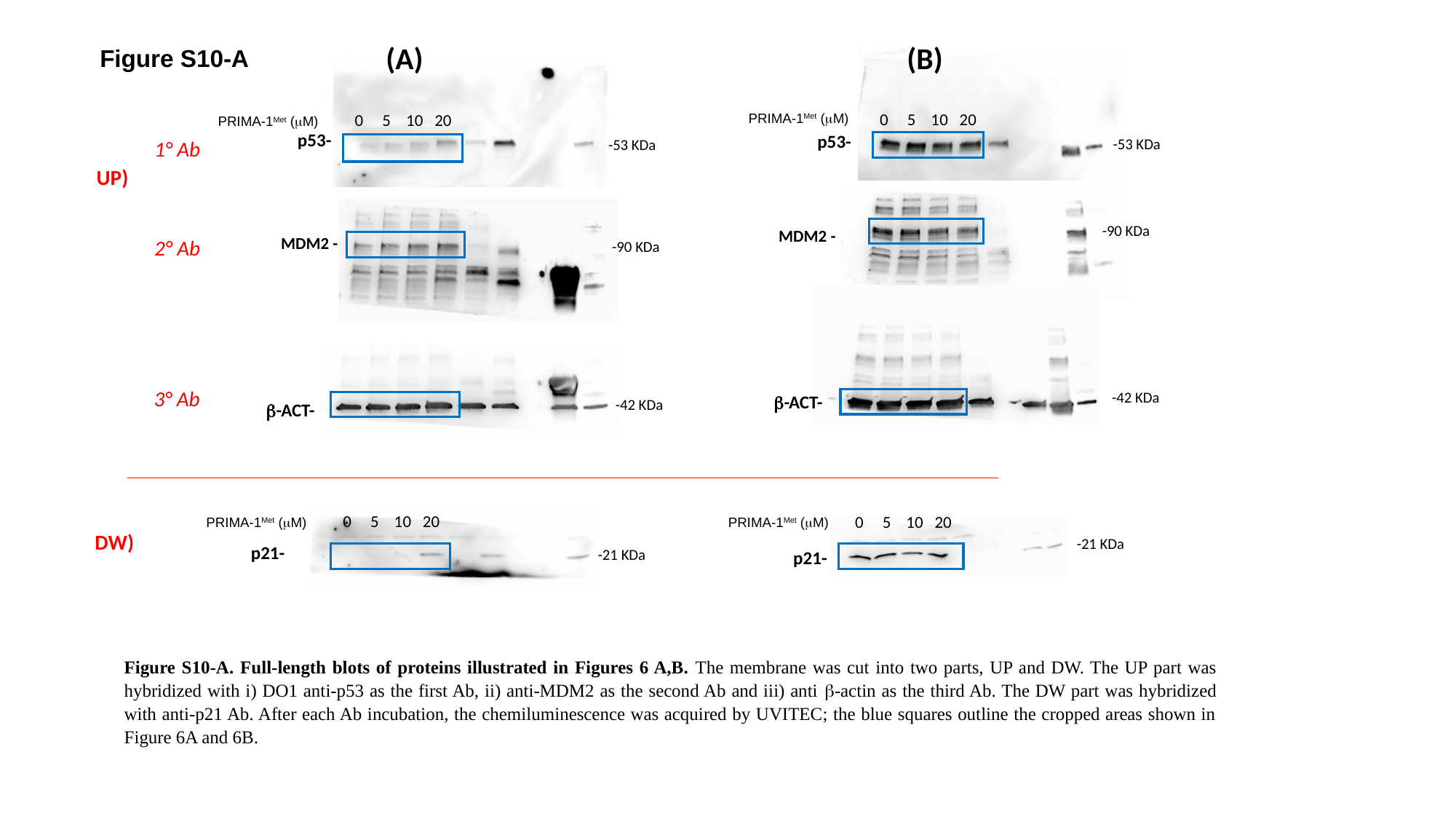

(A)
(B)
Figure S10-A
0 5 10 20
0 5 10 20
PRIMA-1Met (mM)
PRIMA-1Met (mM)
p53-
p53-
-53 KDa
-53 KDa
1° Ab
UP)
-90 KDa
MDM2 -
MDM2 -
2° Ab
-90 KDa
3° Ab
-42 KDa
b-ACT-
-42 KDa
b-ACT-
0 5 10 20
0 5 10 20
PRIMA-1Met (mM)
PRIMA-1Met (mM)
DW)
-21 KDa
p21-
-21 KDa
p21-
Figure S10-A. Full-length blots of proteins illustrated in Figures 6 A,B. The membrane was cut into two parts, UP and DW. The UP part was hybridized with i) DO1 anti-p53 as the first Ab, ii) anti-MDM2 as the second Ab and iii) anti b-actin as the third Ab. The DW part was hybridized with anti-p21 Ab. After each Ab incubation, the chemiluminescence was acquired by UVITEC; the blue squares outline the cropped areas shown in Figure 6A and 6B.

## Slide 15
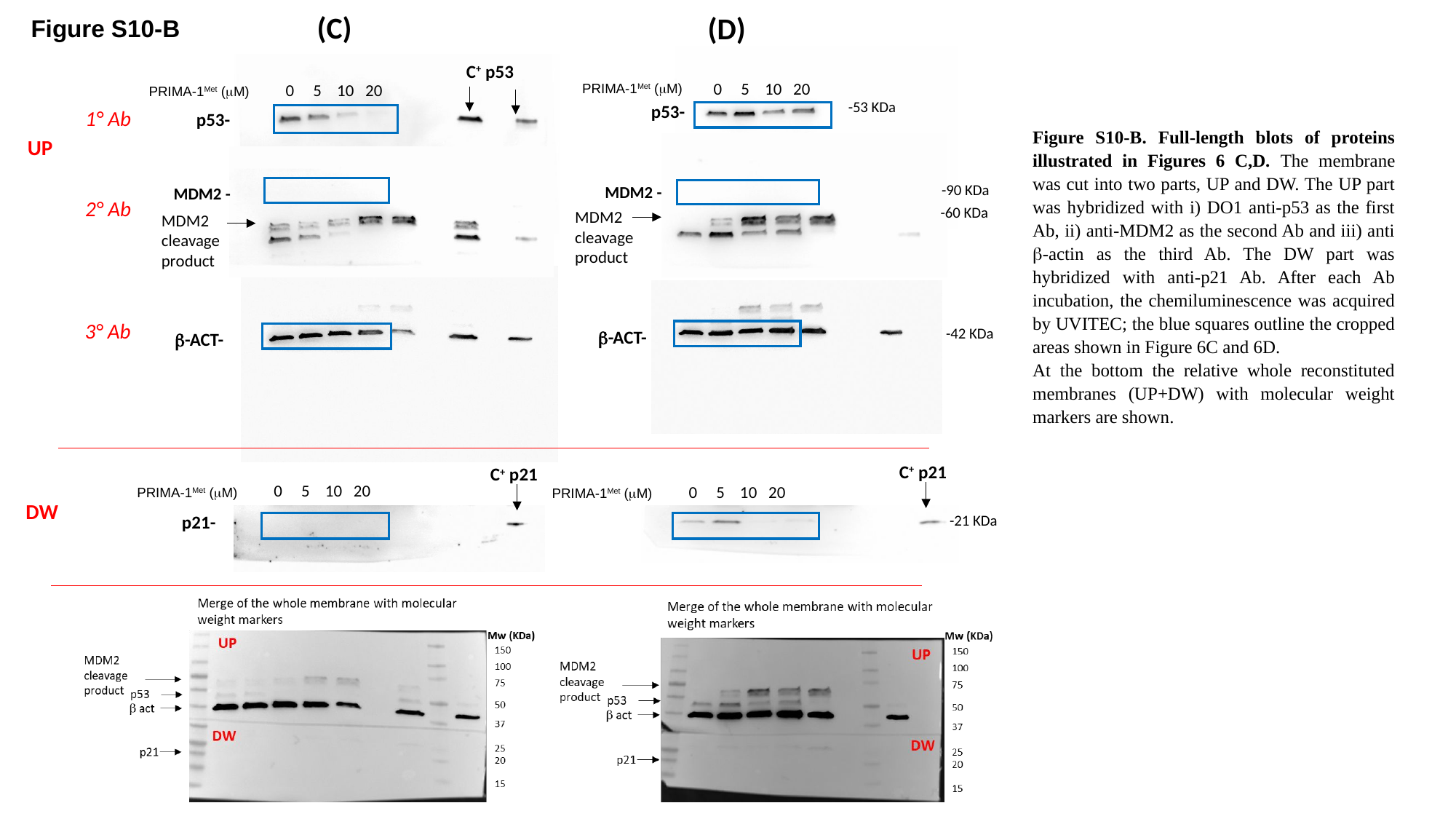

(C)
(D)
Figure S10-B
C+ p53
0 5 10 20
0 5 10 20
PRIMA-1Met (mM)
PRIMA-1Met (mM)
-53 KDa
p53-
1° Ab
p53-
Figure S10-B. Full-length blots of proteins illustrated in Figures 6 C,D. The membrane was cut into two parts, UP and DW. The UP part was hybridized with i) DO1 anti-p53 as the first Ab, ii) anti-MDM2 as the second Ab and iii) anti b-actin as the third Ab. The DW part was hybridized with anti-p21 Ab. After each Ab incubation, the chemiluminescence was acquired by UVITEC; the blue squares outline the cropped areas shown in Figure 6C and 6D.
At the bottom the relative whole reconstituted membranes (UP+DW) with molecular weight markers are shown.
UP
-90 KDa
MDM2 -
MDM2 -
2° Ab
-60 KDa
MDM2 cleavage product
MDM2 cleavage product
3° Ab
-42 KDa
b-ACT-
b-ACT-
C+ p21
C+ p21
0 5 10 20
0 5 10 20
PRIMA-1Met (mM)
PRIMA-1Met (mM)
DW
p21-
-21 KDa

## Slide 16
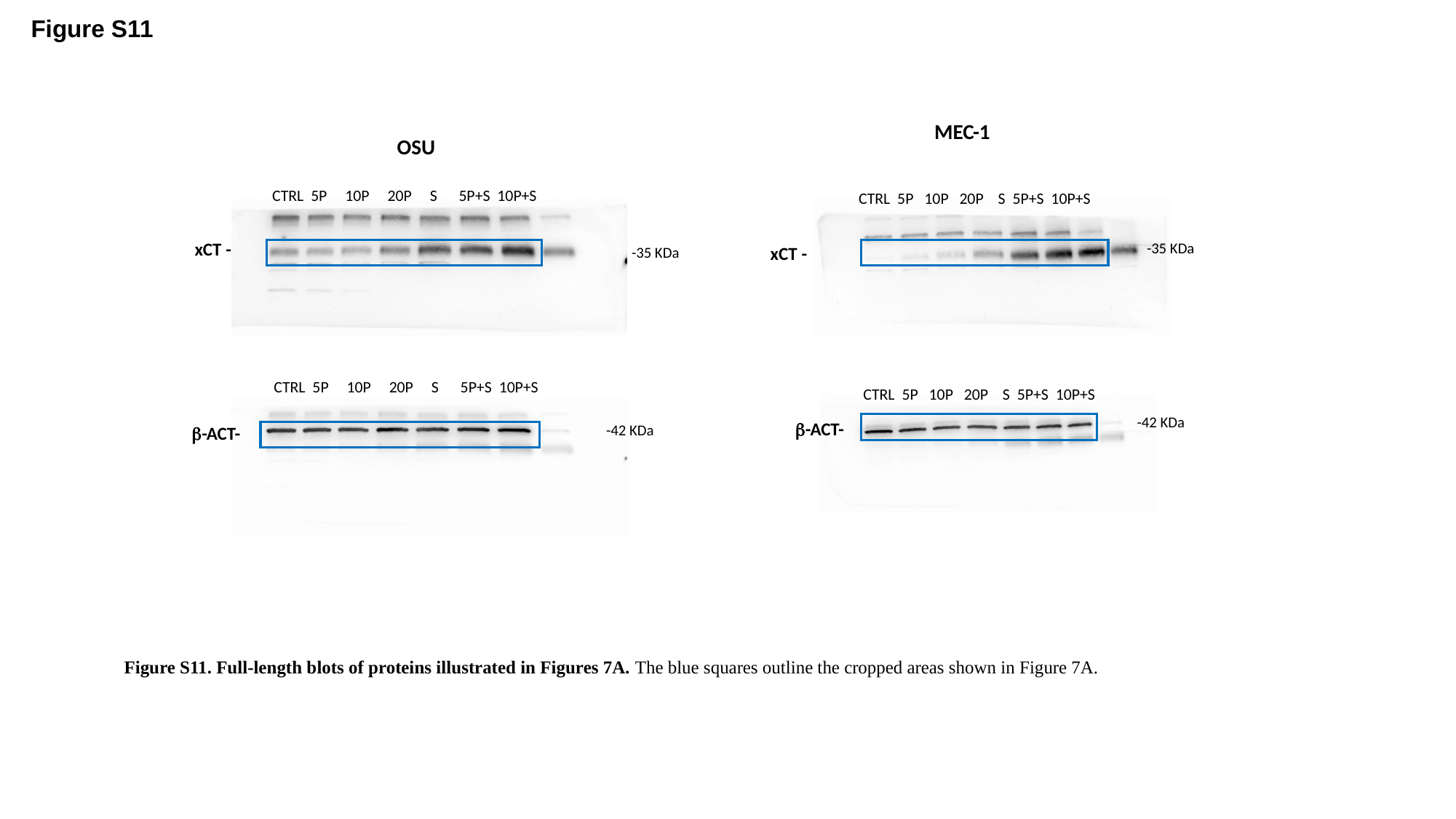

Figure S11
MEC-1
OSU
CTRL 5P 10P 20P S 5P+S 10P+S
CTRL 5P 10P 20P S 5P+S 10P+S
xCT -
-35 KDa
xCT -
-35 KDa
CTRL 5P 10P 20P S 5P+S 10P+S
CTRL 5P 10P 20P S 5P+S 10P+S
-42 KDa
b-ACT-
-42 KDa
b-ACT-
Figure S11. Full-length blots of proteins illustrated in Figures 7A. The blue squares outline the cropped areas shown in Figure 7A.
